# FINDINGS FROM HIGH USERS OF VIDEO TELEHEALTH TO DELIVER OCCUPATIONAL THERAPY SERVICES DURING COVID-19

**DOI:** 10.1093/geroni/igac059.2234

**Published:** 2022-12-20

**Authors:** Megan Gately, Dylan Waller, Emily Metcalf, Lauren Moo

**Affiliations:** VA Bedford Health Care System, Bedford, Massachusetts, United States; VA Portland Health Care System, Portland, Oregon, United States; VA Bedford Health Care System, Bedford, Massachusetts, United States; VA Bedford Health Care System, Bedford, Massachusetts, United States

## Abstract

Occupational therapy (OT) helps older adults improve their ability to perform day-to-day tasks. Veterans Health Administration (VHA) is the single largest employer of occupational therapy (OT) practitioners in the United States and a forerunner in telehealth. As a result of COVID, OT video visits increased by nearly 2000% from 2019 to 2020. To ascertain barriers and facilitators to this shift in care delivery, we conducted interviews between January and April 2021 with OT practitioners (N=27) who were high users of VA Video Connect (VVC), VHA’s videoconferencing software. OT participants were from rural and urban settings, and had completed an average of 536 VVC appointments each in 2020. Participants used VVC to deliver a variety of OT services, including mental health groups and home safety interventions. Facilitators to VVC included, a) Patient characteristics, such as positive perceptions of VVC and technological skill, b) OT clinician characteristics, like flexibility, level of experience, and desire to increase patient access to care, and, c) VHA’s telehealth infrastructure. Barriers included, a) Patients’ lack of familiarity or skills with technology, particularly older patients, b) challenges translating traditionally hands-on care to video, and c) unreliable internet connectivity, particularly for rural patients. This study broadens our understanding of video telehealth service delivery for care which has historically been delivered in brick-and-mortar settings. Understanding challenges and enablers to video telehealth highlights opportunities to increase access to those who face barriers, such as older, rural patients.

